# Antibiotic-Induced Alterations in Gut Microbiota Are Associated with Changes in Glucose Metabolism in Healthy Mice

**DOI:** 10.3389/fmicb.2017.02306

**Published:** 2017-11-22

**Authors:** Richard R. Rodrigues, Renee L. Greer, Xiaoxi Dong, Karen N. DSouza, Manoj Gurung, Jia Y. Wu, Andrey Morgun, Natalia Shulzhenko

**Affiliations:** ^1^Department of Pharmaceutical Sciences, Oregon State University, Corvallis, OR, United States; ^2^Department of Biomedical Sciences, Oregon State University, Corvallis, OR, United States

**Keywords:** antibiotics, gut microbiota, glucose tolerance, lean, non-obese, transkingdom networks

## Abstract

The gut microbiome plays an important role in health and disease. Antibiotics are known to alter gut microbiota, yet their effects on glucose tolerance in lean, normoglycemic mice have not been widely investigated. In this study, we aimed to explore mechanisms by which treatment of lean mice with antibiotics (ampicillin, metronidazole, neomycin, vancomycin, or their cocktail) influences the microbiome and glucose metabolism. Specifically, we sought to: (i) study the effects on body weight, fasting glucose, glucose tolerance, and fasting insulin, (ii) examine the changes in expression of key genes of the bile acid and glucose metabolic pathways in the liver and ileum, (iii) identify the shifts in the cecal microbiota, and (iv) infer interactions between gene expression, microbiome, and the metabolic parameters. Treatment with individual or a cocktail of antibiotics reduced fasting glucose but did not affect body weight. Glucose tolerance changed upon treatment with cocktail, ampicillin, or vancomycin as indicated by reduced area under the curve of the glucose tolerance test. Antibiotic treatment changed gene expression in the ileum and liver, and shifted the alpha and beta diversities of gut microbiota. Network analyses revealed associations between *Akkermansia muciniphila* with fasting glucose and liver farsenoid X receptor (Fxr) in the top ranked host-microbial interactions, suggesting possible mechanisms by which this bacterium can mediate systemic changes in glucose metabolism. We observed *Bacteroides uniformis* to be positively and negatively correlated with hepatic Fxr and Glucose 6-phosphatase, respectively. Overall, our transkingdom network approach is a useful hypothesis generating strategy that offers insights into mechanisms by which antibiotics can regulate glucose tolerance in non-obese healthy animals. Experimental validation of our predicted microbe-phenotype interactions can help identify mechanisms by which antibiotics affect host phenotypes and gut microbiota.

## Introduction

The human gastrointestinal tract contains a multitude of microbiota, including bacteria, viruses, and fungi (Utzschneider et al., 2016). Their genome, although variable between individuals (Human Microbiome Project Consortium, 2012), is capable of a diverse set of functions that may influence the host’s metabolic and immune systems (Tremaroli and Backhed, 2012; Greer et al., 2013; Sanz et al., 2015), including normal homeostasis (Utzschneider et al., 2016). Changes in the gut microbes have recently been associated with various diseases (Qin et al., 2012; Karlsson et al., 2013; Wu et al., 2015). For example, changes in Lactobacillus, Clostridium, Ruminococcus sp., *E. coli*, Bacteroides, *Akkermansia muciniphila* are observed in diabetic and obese patients (Qin et al., 2012; Karlsson et al., 2013; Murri et al., 2013; Chakraborti, 2015; Kasai et al., 2015; Sanz et al., 2015). These diverse results indicate a need for a better understanding of the mechanistic roles specific taxa play in the regulation of host metabolic functions.

Antibiotics add an interesting dynamic to the host-microbiome relationship. Although, antibiotics are well-known to cause short (Perez-Cobas et al., 2013; Pallav et al., 2014; Panda et al., 2014) and long-term (De La Cochetiere et al., 2005; Jernberg et al., 2007; Dethlefsen et al., 2008; Jakobsson et al., 2010; Dethlefsen and Relman, 2011; Raymond et al., 2016) alterations in the gut microbiome, there is a lack of consensus on their effects on glucose tolerance, body weight and other metabolic parameters (Francino, 2015; Mikkelsen et al., 2016). Moreover, effects of antibiotics in lean, normoglycemic mice as compared to mouse obesity models have not been widely investigated. An intervention study in healthy, glucose tolerant young human males treated with 4-days broad-spectrum antibiotics cocktail showed shifts in the cultivable gut microbiota but no changes in postprandial plasma glucose and serum insulin (Mikkelsen et al., 2016). Due to the use of a broad-acting antibiotic cocktail in a short course as well as the use of fecal samples for culture-based bacterial assessment, this study provides limited insight on a comprehensive picture of changes in intestinal microbes and on associations between individual antibiotics and specific intestinal microbes. Understanding antibiotic-microbiome interactions and their effects on glucose metabolism in healthy mammals is critical for identifying initial changes in microbiota that eventually may lead to diseases such as obesity and diabetes.

In this study, we aimed to understand the regulatory mechanisms by which individual antibiotics and their cocktail influence the cecal microbiome and host phenotypes in lean mice, namely, gene expression and metabolic parameters. By treating lean mice with different antibiotics we sought to: (i) study the effects on body weight, fasting glucose, glucose tolerance, and fasting insulin, (ii) examine the changes in expression of key genes of the bile acid and glucose metabolic pathways in the liver and ileum, (iii) identify the shifts in the cecal microbiota, and (iv) infer interactions between gene expression, microbiome, and the metabolic parameters. We repeated the entire experiment twice and performed meta-analyses to increase the confidence of our results.

## Materials and Methods

### Mice and Antibiotics Treatment

Eight weeks old adult male Swiss Webster mice were initially purchased from Taconic Biosciences (Germantown, MD, United States). Mice were housed at the Laboratory Animal Resource Center at Oregon State University for 3–5 days for acclimation under standard 12-h light cycle with free access to food (5001, Research Diets) and water. Experimental procedures were carried out in accordance with protocols approved by the Oregon State University Institutional Animal Care and Use Committee. Mice were given single, cocktail, or no antibiotics for 4 weeks to create a stable altered microbiome. Antibiotics were administered in autoclaved drinking water individually, or in a cocktail for 4 weeks in the following concentrations: ampicillin (1 gl^-1^), metronidazole (1 gl^-1^), neomycin trisulfate (1 gl^-1^), and vancomycin (0.5 gl^-1^). This time course is consistent with standard antibiotic administration used in multiple studies for altering microbiota (Rakoff-Nahoum et al., 2004; Morgun et al., 2015; Greer R.L. et al., 2016). Each group consisted of five mice per experiment, total 30 mice per experiment, except for four mice in the cocktail group from the second experiment. Water consumption was monitored over the 4 weeks treatment period and all groups showed consumption equivalent to control water.

### Glucose Tolerance Testing

Mice were fasted for 6 h during the light phase with free access to water. A concentration of 2 mg kg^-1^ glucose (Sigma–Aldrich) was injected intraperitoneally. Blood glucose was measured at 0 (immediately before glucose injection), 15, 30, 60, and 120 min with a Freestyle Lite glucometer (Abbot Diabetes Care).

### Serum Collection and Hormone Measures

Mice were fasted for 6 h during the light phase with free access to water. Serum was collected via submandibular bleed using BD microtainer serum separator tubes. Fasting insulin was measured by ultrasensitive ELISA (Crystal Chem) according to manufacturer’s protocols.

### Bacterial DNA Extraction, 16S rRNA Gene Library Preparation and PCR

Unflushed cecal tissue and content was suspended in 1.4 ml ASL buffer (Qiagen) and homogenized with 2.8 mm ceramic beads followed by 0.5 mm glass beads using an OMNI Bead Ruptor (OMNI International). DNA was extracted from the entire resulting suspension using QIAamp DNA Stool Mini Kit (Qiagen) according to manufacturer’s protocol. DNA was quantified using Qubit broad range DNA assay (Life Technologies). The V4 region of 16s rRNA gene was amplified using universal primers (515f and 806r) (Caporaso et al., 2012). Individual samples were barcoded, pooled to construct the sequencing library, and then sequenced using an Illumina Miseq (Illumina, San Diego, CA, United States) to generate pair-ended 250 nt reads. Quantitative PCR was performed for *A. muciniphila* as described in Schneeberger et al. (2015) with DNA for standard curve isolated from the cultivated microbe.

### RNA Preparation and Gene Expression Analysis

Liver and ileum (flushed out of content) were collected and snap frozen prior to RNA extraction. Liver was homogenized using OMNI Rotor-Stator Homogenizer in Trizol and RNA was extracted using Trizol/chloroform extraction followed by the RNeasy Mini kit (Qiagen). Ileum RNA was extracted using OMNI Bead Ruptor and 2.8 mm ceramic beads (OMNI International) in RLT buffer followed by Qiashredder and RNeasy kit using Qiacube (Qiagen) automated extraction according to manufacturer’s specifications. Total RNA was quantified using Nanodrop (Thermo Scientific). Complementary DNA was prepared using iScript reverse transcription kit (Bio-Rad) and qPCR was performed using QuantiFast SYBR mix (Qiagen) and StepOne Plus Real Time PCR system and software (Applied Biosystems). Primers used for qPCR are listed in **Supplementary Table S1**.

### Statistical Analysis of Phenotypic Data

An outlier value per group per experiment was removed (if *p*-value < 5%) for each phenotype (metabolic parameters and genes) using the default Grubb’s test from R package *outliers* v0.14 (Komsta, 2011). The data was log_2_ transformed and differential phenotypes (antibiotics vs. control) were detected using *limma* (Ritchie et al., 2015) (Bioconductor 3.4, BiocInstaller 1.24.0, R 3.3.2) per experiment. A combined Fisher’s *p*-value was calculated for each phenotype from the *p*-values for the limma t-statistic from each experiment. A false discovery rate (FDR) was calculated on the combined *p*-values. Change in phenotype was considered statistically significant if the phenotype had same direction of (abx/control) fold change in both experiments, individual *p*-value < 20% in each experiment, Fisher’s combined *p*-value (Fisher, 1932) <5% and FDR < 10%. The dot plots for the phenotypes were generated using R package *ggplot2* (Wickham, 2009) and the GTT curves were generated using GraphPad Prism software v7.03.

### Analyses of 16S rRNA Gene Sequencing Data

The samples were demultiplexed and forward-end fastq files were analyzed using QIIME v. 1.9.1 (Caporaso et al., 2010). The default quality filter parameters from QIIME’s *split_libraries_fastq.py* were applied to retain high quality reads (Phred quality score > = 20 and minimum read length = 75% of 250 nucleotides). A closed reference OTU picking with 97% sequence similarity was performed using UCLUST (Edgar, 2010) and Greengenes reference database v13.8 (DeSantis et al., 2006; McDonald et al., 2012) to cluster 16S rRNA gene sequence reads into OTUs and assign taxonomy. The reference sequence of an OTU from the Greengenes database was used to obtain species level taxonomic assignment using Megablast (Altschul et al., 1997; Morgulis et al., 2008) (top hit using default parameters). A threshold of 99% cumulative abundance across all samples in an experiment was used to retain abundant microbes, thus removing OTUs with approximately <0.01% abundance across all samples in that experiment. The read counts were normalized using cumulative sum scaling (Paulson et al., 2013) followed by quantile normalization.

The normalized OTU tables were used for diversity and statistical analysis. Briefly, a sampling depth of 200,000 sequences per sample was used for rarefaction. The alpha diversity metrics were calculated on unrarefied and rarefied OTU tables (**Supplementary Table S2**). The Shannon diversity index (from rarefied data) for samples with and without antibiotics treatment was compared with a non-parametric *t*-test. The difference was considered to be statistically significant if the direction of (abx/control) fold change in both experiments is the same, individual *p*-value < 2% in each experiment, Fisher’s combined *p*-value < 0.1% and FDR < 0.1%. Beta diversity was calculated using weighted UniFrac (Lozupone and Knight, 2005) and the distances were used for PCoA (Gower, 1998) and visualized using EMPeror (Vazquez-Baeza et al., 2013). The taxonomic summary bar plots were used to visualize abundance at the phylum and order levels.

The log_2_ transformed OTU tables were used for limma analysis. Meta-analysis was performed using the same criteria as applied for phenotypes to identify differentially abundant OTUs. A heatmap with row scaling was generated for each experiment using R packages *ggfortify* v0.2 (Horikoshi and Tang, 2016) and *gplots* v3.0.1 (Warnes et al., 2016). Hierarchical clustering was used to group OTUs (rows) based on similar abundance patterns across the groups in the first experiment and the same row order was used for the second experiment without row-wise clustering.

#### Network Reconstruction and Prioritizing Microbe-Phenotype Edges

Spearman rank correlations were calculated between all pairs of genes, microbes, and metabolic parameters across all samples or per-group in an experiment. A combined Fisher’s *p*-value was calculated for each pair from the *p*-values for the correlation from each experiment. A FDR was calculated on the combined *p*-values separately for the following correlations: (i) within genes, (ii) within metabolic parameters, (iii) between genes and metabolic parameters, and (iv) between OTUs and phenotypes (genes or metabolic parameters).

We retained edges that satisfy the following criteria: the sign of correlation coefficients in the two experiments should be consistent, individual *p*-value of correlation within each experiment is <20%, combined Fisher’s *p*-value of all experiments <5% and FDR cutoff of 10% for edges without a microbial node (i, ii, and iii), whereas 1% for edges containing at least one microbial node (iv).

Next, the transkingdom network was generated (Dong et al., 2015; Morgun et al., 2015; Greer R.L. et al., 2016; Rodrigues et al., 2017) by keeping the criteria-satisfying phenotypic (i, ii, and iii) and OTU-phenotype (iv) edges, where the OTU has >0.5% median abundance across the two experiments in at least one group.

Finally, an OTU-phenotype edge was retained if it showed consistent sign of per-group Spearman correlation coefficient between the two experiments, principles of causality compliancy (Yambartsev et al., 2016) [i.e., satisfied fold change relationship between the two partners in the appropriate (abx vs. control) comparison] in at least one group, and the same sign of correlation coefficient across different groups. To put this bipartite network in perspective of the phenotypic connections a phenotypic edge was included (only during visualization) if its strength of correlation was stronger than at least one OTU-phenotype edges connecting the phenotypes. Network topology statistics, namely degree and betweenness centrality (BC), were calculated using NetworkAnalyzer (Assenov et al., 2008) in Cytoscape v3.5 (Shannon et al., 2003). These edges were ranked using a score of maximum (per-group OTU abundance) × absolute [median (per-group correlation)] to prioritize OTUs and the phenotypes they potentially affect, where the per-group OTU abundance and correlation are medians across the two experiments. The top hit of BLAST for the Greengenes representative sequence for an OTU was used to obtain species level identification.

### Data Availability

Raw reads of 16S rRNA gene sequencing have been deposited at NCBI under BioProject PRJNA394608, Biosamples of SAMN07356206 – SAMN07356264, Sequence Read Archive SRP112596.

## Results

Lean, normoglycemic male mice were left untreated, or were treated with ampicillin, metronidazole, neomycin or vancomycin, or a cocktail containing all four antibiotics for 4 weeks to study the effects of antibiotic treatment on glucose tolerance, genes involved in glucose and bile acid metabolism, and the gut microbiota. Antibiotics resulted in different patterns of changes in the metabolic parameters, gene expression, and intestinal microbiome.

### Antibiotics Improved Glucose Tolerance in Lean Mice

**FIGURE 1:**
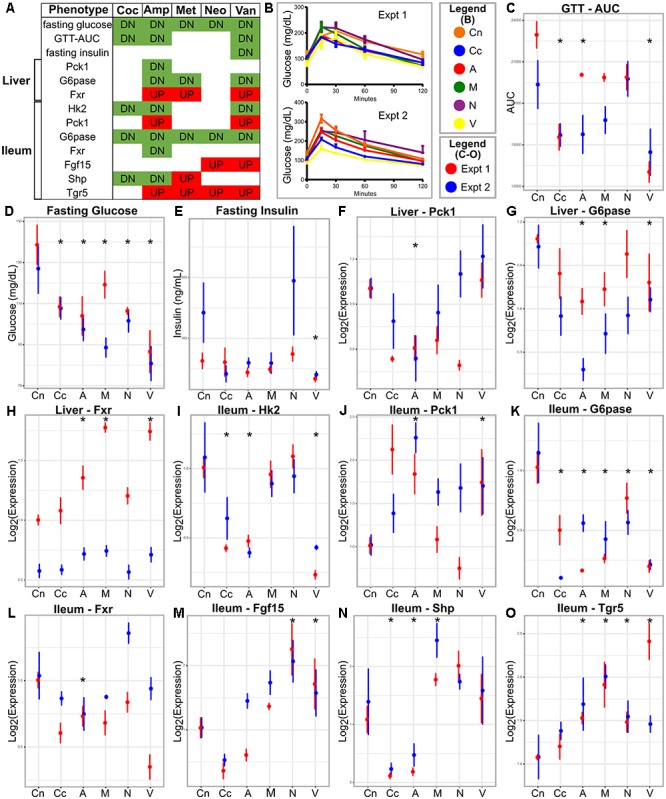
Metabolic parameters and gene expression in antibiotic-treated and control animals. **(A)** Summary table; the red and green colors indicate increase and decrease, respectively, in antibiotic treated group compared to the control. **(B)** GTT curves for the antibiotics treated and control groups in the two experiments. **(C–E)** Metabolic parameters and gene **(F–O)** expression represented as means with standard error bars. The red and blue colors indicate experiments one and two, respectively. Asterisks indicate parameters that show statistically significant differences upon antibiotics treatment compared to untreated control mice [same direction of (abx/control) fold change in both experiments, individual *p*-value < 20% in each experiment, Fisher’s combined *p*-value < 5% and FDR < 10%]. Cn, Control; Coc or Cc, cocktail; Amp or A, ampicillin; Met or M, metronidazole; Neo or N, neomycin; Van or V, vancomycin.

No metabolic parameter worsened following antibiotics treatment (**Figure 1** and **Supplementary Figure S1**). We observed that treatment with individual or cocktail of antibiotics reduced fasting glucose, but did not change body weight. Glucose tolerance improved upon treatment with cocktail, ampicillin, or vancomycin as indicated by reduced AUC of the GTT. Treatment with all antibiotics, including metronidazole or neomycin reduced fasting glucose levels, however, the latter two did not cause changes in systemic glucose tolerance. Fasting insulin was reduced only when the mice were treated with vancomycin. Overall, glucose metabolism was regulated by antibiotic treatment.

### Antibiotics Changed Expression of Genes Involved in Glucose and Bile Acid Metabolism

Tissue specific host gene expression is important in many metabolic processes (Thomas et al., 2008; Chiang, 2013) and regulated by gut microbiota (Larsson et al., 2012). These, along with the knowledge that intestinal glucose metabolism can control systemic glucose levels (Saeidi et al., 2013), led us to examine the expression of key glucose and bile acid metabolic genes in the liver and the ileum.

The majority of the tested genes in the ileum showed changes in expression due to antibiotic treatment (**Figure 1**). Ileum Hk2 and G6pase transcripts showed decreased expressions after treatment with cocktail, ampicillin, or vancomycin. Ileum Pck1 and Tgr5 mRNA were increased after treatment with ampicillin or vancomycin, but showed no changes after with cocktail. Ileal Hk1 and Glut1 did not change gene expression after antibiotics, whereas, Fgf15, Fxr, and Shp showed antibiotic-specific patterns in expression.

Only three genes showed differential expression in the liver following antibiotic treatment (**Figure 1**). Fxr and G6pase showed increased and decreased expression, respectively, in ampicillin or vancomycin treated mice. Pck1 showed lower expressions in ampicillin treated samples. Hk2 and Insr genes in the liver did not change following antibiotics treatment.

**FIGURE 2:**
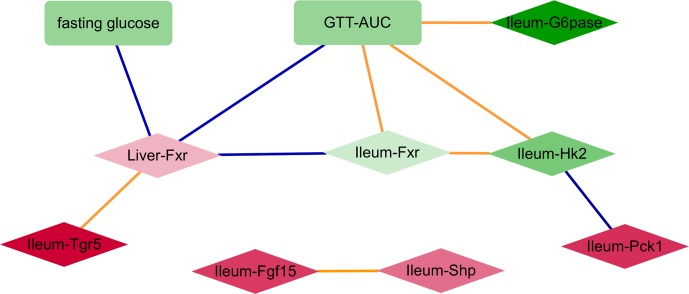
A network consisting of metabolic parameters and gene expression from liver or ileum. An edge indicates the sign of spearman correlation coefficients across all samples in the two experiments are consistent, individual *p*-value of correlation within each experiment is <20%, Fisher’s combined *p*-value of all experiments <5% and FDR < 10%. Red and green colors indicate increased and decreased median fold change (abx/control) for nodes, respectively; where the color intensity corresponds to the level of fold change (e.g., dark color indicates fold change ratio is further away from 1); diamond and rectangle shapes indicate genes and metabolic parameters, respectively. Blue and orange colors indicate negative and positive correlated edges, respectively.

Despite some variability in tissue specific behavior of genes in response to antibiotics, the improved glucose tolerance upon antibiotic treatment suggests that relationships between gene expression and metabolic parameters are mostly preserved across all groups. Hence, we constructed a correlation network consisting of (differentially expressed) genes and (differentially abundant) metabolic parameters using all samples per experiment (**Figure 2**). Genes from the ileum, including G6pase, Hk2, and Fxr were strongly connected with the GTT-AUC. The Fxr gene in the liver was positively correlated with the ileum Tgr5 but negatively correlated with ileum Fxr and with fasting glucose and GTT. Altogether, this network indicates opposite effects of intestinal and liver Fxr on glucose metabolism. Furthermore, it also suggests that increased glycolytic gene expression program in ileum is connected to worsening of systemic glucose metabolism.

### Antibiotics Caused Shifts in Microbial Communities

Microbiome composition is known to be affected by antibiotics (De La Cochetiere et al., 2005; Jernberg et al., 2007; Dethlefsen et al., 2008; Jakobsson et al., 2010; Dethlefsen and Relman, 2011; Perez-Cobas et al., 2013; Pallav et al., 2014; Panda et al., 2014; Raymond et al., 2016) and involved in metabolic processes (Larsson et al., 2012; Tremaroli and Backhed, 2012; Sanz et al., 2015; Utzschneider et al., 2016), so we hypothesized that gut microbes might play a mechanistic role in the effect of antibiotics (Morgun et al., 2015; Greer R. et al., 2016; Greer R.L. et al., 2016) on host glucose metabolism (Caesar et al., 2012; Greer R.L. et al., 2016). Sequencing the 16S rRNA gene of the cecal microbiome from the two experiments provided a total of 14,321,948 high quality reads with mean length of 248.50 bases and standard deviation of 9.42. A threshold of 99% cumulative abundance across all samples per experiment retained 734 and 677 OTUs in the two experiments (overlap of 561 OTUs) with 5,450,867 and 5,525,927 assigned sequences to the OTUs. The alpha diversity metrics on the normalized and rarefied OTU tables are provided in **Supplementary Table S2**. As expected, the cocktail of antibiotics reduced the diversity of the samples compared to untreated or individual antibiotics (**Supplementary Figures S2**, **S3**). Shannon diversity comparisons showed that alpha diversity decreased when treated with cocktail, ampicillin, or vancomycin (**Supplementary Figure S2**).

**FIGURE 3:**
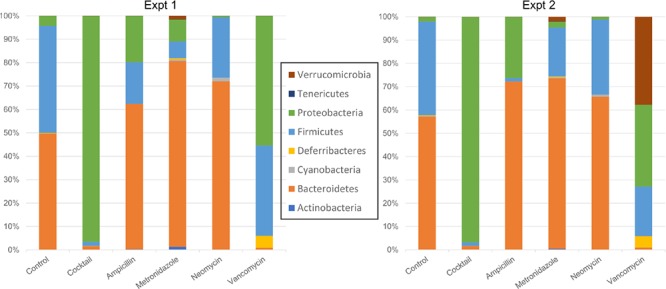
Taxonomic plots showing mean bacterial abundance across the different groups at the phylum level.

**FIGURE 4:**
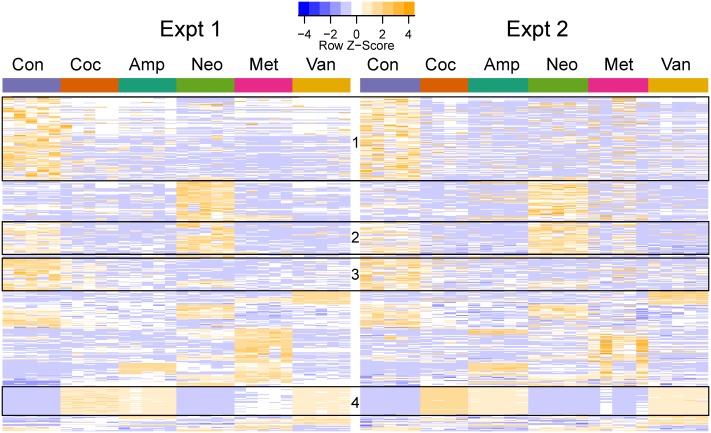
Heatmap of differentially abundant OTUs in the different groups. The represented OTUs passed the same statistical criteria as that used for phenotypes. The rows (OTUs) were clustered based on the abundance in the first experiment and the same row order was used for the second experiment. The rows were scaled, and orange and blue colors indicate increased and decreased abundance, respectively. The ids and taxa names for the rows (OTUs) are provided in **Supplementary Table S3**. The boxes indicate OTU clusters with the same taxonomy: Boxes 1 (113/117), 2 (43/46), and 3 (43/45) primarily contained Clostridiales (phylum: Firmicutes), while Box 4 (38/38) had Enterobacteriales (phylum: Proteobacteria).

A PCoA analysis using the weighted UniFrac suggested that the overall community composition from vancomycin and ampicillin treatment was closer to that when treated with antibiotics cocktail (**Supplementary Figure S3**). At the phylum level, Firmicutes decreased in cocktail, ampicillin, and metronidazole treated samples. Bacteroidetes decreased upon cocktail and vancomycin treatment but increased when treated with metronidazole (**Figures 3**, **4** and **Supplementary Figure S5**; Sheet in **Supplementary Table S3**). The treatment with antibiotics showed similar patterns of change in the abundant bacteria at the order level, while less abundant bacteria showed antibiotic specific changes (**Supplementary Figures S4**, **S6**). Vancomycin treatment increased Verrucomicrobiales in both experiments compared to control, however, increase in the second experiment was extremely high (fold change = 17,480) compared to the first (fold change = 358). Of note, this order was presented by single member (*A. muciniphila*). Thus, we also analyzed the abundance of this microbe via specific PCR and confirmed differences between two experiments in vancomycin treated groups (0.04 and 9794.4 ng DNA *A. muciniphila*/ g cecal content in the first and second experiments, respectively).

Cocktail, ampicillin, and vancomycin treated samples showed similar patterns of change at the OTU level as compared to the microbiome of control samples (**Figure 4**), which may be related to the fact that only these antibiotic treatments were able to change GTT-AUC (**Figure 1**). *Prevotella* sp. (OTU_189721) was the most abundant OTU in control (median abundance across two groups (24%), neomycin (38%), and metronidazole (17.5%) treated samples. Enterobacteriaceae family (OTU_1111294) was the most abundant in cocktail (38%) and vancomycin (28%), and the third most abundant in ampicillin (14%) treated samples. Bacteroides uniformis (OTU_589071) was the most abundant upon ampicillin treatment (22.8%), while *A. muciniphila* was the second most abundant in vancomycin treated samples (17.5%) (**Supplementary Table S4**).

### Microbes Are Associated with Changed Phenotypes

Gut microbiota can control the expression of many genes in the small intestine (Larsson et al., 2012). Therefore, we asked whether the antibiotic-induced changes in the microbiome were potentially connected to the observed changes in gene expression. We constructed a transkingdom network using all groups, consisting of genes, metabolic parameters, and OTUs, to identify candidate interactions whereby microbes can mediate changes in systemic glucose tolerance and found 131 OTU-phenotype edges.

**FIGURE 5:**
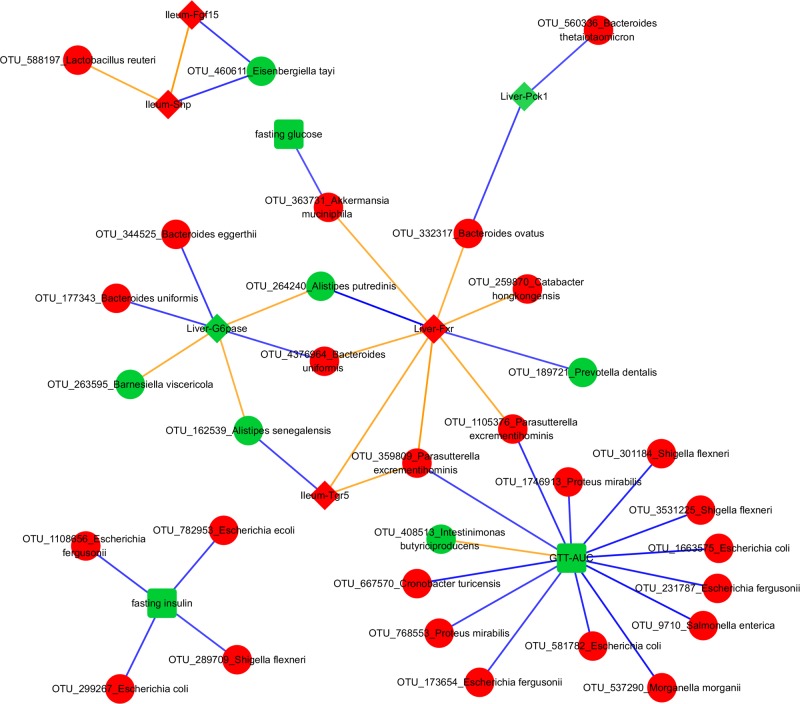
Transkingdom Network. Red and green colors indicate increased and decreased median fold change (abx/control) for nodes, respectively; diamond, rectangle, and circle shapes indicate genes, metabolic parameters, and OTUs, respectively. Blue and orange colors indicate negative and positive correlated edges, respectively. We indicate a phenotypic edge if its strength of correlation (in the phenotypic network) is stronger than at least one OTU-phenotype edges connecting the phenotypes.

**FIGURE 6:**
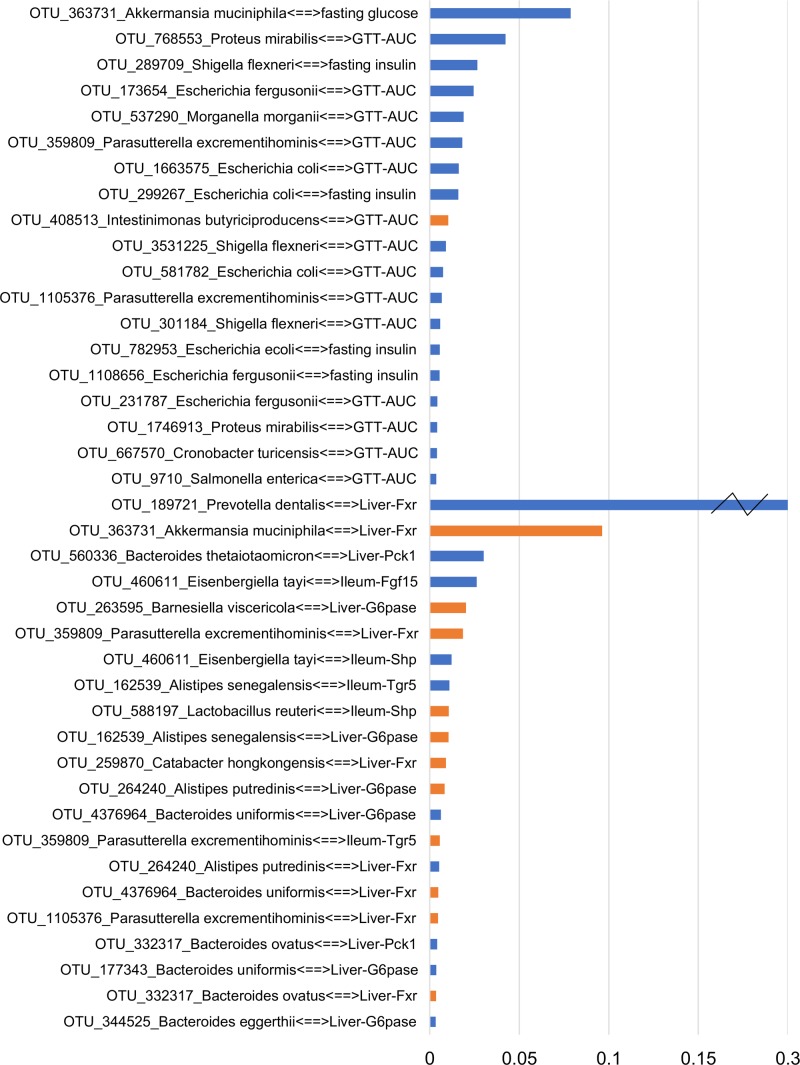
Ranking the transkingdom edges. The score accounts for the bacterial abundance and its correlation strength with a phenotype. Blue and orange colors indicate negative and positive correlated edges, respectively. A broken axis (indicated by “Z”) allows for clear visualization of the other pairs.

To focus on microbe-phenotype relationships that are not affected by type of antibiotics, we retained the 40 edges (**Figure 5**) that maintained the same sign of correlation coefficient between the various groups of both experiments and consistent with potential causal relations (Dong et al., 2015; Morgun et al., 2015; Greer R.L. et al., 2016; Rodrigues et al., 2017) in at least one group of both experiments. Overall, this means that while a strength of OTU-phenotype interaction may be weak for a particular antibiotic group, this interaction may still be important in mediating effects of antibiotics on the host in general. The abundance of a microbe and its strength of correlation with a phenotype are expected to be crucial in mediating the effects, hence these 40 edges were ranked using a score that takes into account the maximum per-group OTU abundance and the median per-group correlation strength with a phenotype (**Figure 6**; See formula in section “Materials and Methods”).

NCBI BLAST on an OTU’s Greengenes reference sequence was used to obtain its (closest) species level identification. Interestingly, associations between *A. muciniphila* with fasting glucose and liver Fxr showed as the top interactions suggesting a possible mechanism through which this bacterium can mediate systemic changes in glucose metabolism. *Proteus mirabilis* was negatively correlated with GTT-AUC. *Bacteroides uniformis* was positively and negatively correlated with hepatic Fxr and G6pase, respectively. Importance of phenotypes in the network was also determined by degrees of connectedness (degree) and BC. GTT-AUC (degree = 14, BC score = 0.65) and liver Fxr (degree = 8, BC score = 0.60) were the highly connected metabolic parameter and gene, respectively, as well as the key nodes in the largest connected component of the network. Overall, it suggests that gut microbiota potentially influences the liver metabolic genes and systemic metabolic parameters and mediates the effects of antibiotics on host phenotypes.

## Discussion

Germ-free Swiss Webster mice showed improved glucose metabolism (Caesar et al., 2012), suggesting that microbiota regulate metabolism in this strain. Furthermore, Swiss Webster mice are traditionally outbred, so more similar to human population. Therefore, Swiss Webster mice were selected for this study. While research has been done to study the effects of antibiotics on microbiota and glucose tolerance in diseased models (Francino, 2015), these effects in lean, non-diabetic or normoglycemic mice are not well studied. Such a study can provide meaningful insights into the host-microbial interactions and consequences of antibiotics in healthy population, and may allow the prediction of protective mechanisms and risk factors for development of diabetes.

To the best of our knowledge, our study is the first to show the ability of antibiotics to change glucose metabolism in healthy mice. The reduced fasting glucose and GTT-AUC in two experiments, especially in the ampicillin, vancomycin, and cocktail treated samples suggest that antibiotic treatment cause systemic improvements in glucose tolerance. Although our observations of reduced GTT-AUC contradicts with the absence of change observed in healthy humans (Mikkelsen et al., 2015), the cocktail ingredients and time course of antibiotic treatment (1 week vs. 4 weeks) of the two studies may be more critical factors contributing to this disagreement than differences between two species (i.e., mice and humans). Additionally, the unchanged insulin secretion by cocktail treatment in our study is in agreement with the study performed in humans (Mikkelsen et al., 2015). Noteworthy, one study did not observe any changes in fasting glucose and insulin in chow fed C57BL6 mice when treated with broad-spectrum antibiotics (ampicillin, metronidazole, and neomycin) (Pang et al., 2013). This disagreement might be due to differences in the mice strain, gut bacterial communities in different mouse facilities and the antibiotics used in the cocktail. For example, effect of vancomycin on glucose metabolism can be partially attributed (at least for our second experiment) by increased abundance of *A. muciniphila* which is missing in some mouse colonies. While the two studies (Pang et al., 2013; Mikkelsen et al., 2015) have some discrepancies with our observations, there are numerous supportive studies using germ-free (Caesar et al., 2012) or diet-induced obese (Cani et al., 2008, 2014; Membrez et al., 2008; Carvalho et al., 2012; Hwang et al., 2015; Fujisaka et al., 2016) mice that have shown improved glucose tolerance in the absence of microbiota and with antibiotics usage and the consequently modulated microbiota.

The expression of key genes from the glucose and bile acid metabolism pathways were measured, since bile acid signaling plays an important role in glucose homeostasis (Nguyen and Bouscarel, 2008; Trauner et al., 2010; Prawitt et al., 2011; Chiang, 2013; Nie et al., 2015; Trabelsi et al., 2016). We observed well-known (and therefore expected) relationships between the tissue-specific expression patterns of different genes themselves and with the systemic metabolic parameters in vancomycin and ampicillin. Low hepatic Fxr causes increased gluconeogenesis (Ma et al., 2006) and bile acid synthesis (Duran-Sandoval et al., 2004), while increased liver Fxr (Li and Guo, 2015) and intestinal Fgf15 (Holt et al., 2003) suppress hepatic bile acid synthesis (Kong et al., 2012) and regulate hepatic glucose metabolism (Potthoff et al., 2011). Also, increased liver Fxr represses G6pase (Yamagata et al., 2004; Ma et al., 2006; Zhang et al., 2006), Pck1 (De Fabiani et al., 2003; Yamagata et al., 2004; Ma et al., 2006), and like repressed ileum Fxr (Jiang et al., 2015b), improves glucose tolerance (Ma et al., 2006; Zhang et al., 2013), similar to our results. Along the same lines, mice treated with Fgf15 showed improved glucose metabolism (Zhou et al., 2017) and increased intestinal Fgf15 expression represses liver G6pase and Pck1, key enzymes for liver gluconeogenesis (Potthoff et al., 2011). In line with our observations in vancomycin treatment, Fxr agonist obeticholic acid (OCA; Intercept Pharmaceuticals, New York, NY, United States) increased mRNA levels of Fgf15 and Tgr5 in the ileum of C57BL/6J mice without increase in ileum Fxr (Pathak et al., 2017), and an increase in ileum Fgf15 expression decreased plasma glucose levels even with low insulin levels (Potthoff et al., 2011). Supporting our results from ampicillin treatment, a study showed that treating mice on high fat diet with antibiotics cocktail inhibited Fxr signaling in the ileum but not in the liver, and observed decreased expression of Shp in the ileum (Jiang et al., 2015a). Furthermore, Fxr and Shp mRNA in the ileum were also reduced in germ-free Swiss Webster mice on chow diet compared to the conventionally raised (untreated) group (Sayin et al., 2013). Overall, these studies along with ours support the idea that bile acids repress gluconeogenesis (Modica et al., 2010). However, it is interesting to see that while metronidazole, neomycin, and cocktail do not show the above changes in gene expression there is still improvement in fasting glucose and/or glucose tolerance, suggesting that microbiota might play an even bigger role in mediating the effects of antibiotics on phenotypes through additional mechanisms not explored here.

In fact, microbiota can change the expression of many genes in the ileum (Larsson et al., 2012). Their study observed down-regulated Hk2, G6pase, and Shp in the ileum of germ-free mice when compared to conventionally raised mice on chow diet, supporting our results from cocktail (ampicillin, or vancomycin) treatment. Also, some of the changes in gut microbiota to antibiotics that we observe in our data, e.g., the increased Verrucomicrobiales following vancomycin (Hansen et al., 2012) and Enterobacteriales increase upon treatment with ampicillin or vancomycin (Ubeda et al., 2010), are well documented.

While the effects of microbes on systemic glucose tolerance in lean subjects are rarely studied, their ability to influence glucose metabolism is well-recognized (De Vadder et al., 2016). A good example of well-established causal relations between specific bacteria and glucose metabolism is beneficial effect of *A. muciniphila*. For example, it was shown that *A. muciniphila* was able to delay the onset of diabetes in the vancomycin treated mice (Hansen et al., 2012). Furthermore, multiple studies demonstrated that this bacterium can improve glucose metabolism in animal models and in humans (Everard et al., 2013; Zhang et al., 2013; Joyce and Gahan, 2014; Shin et al., 2014; Anhe et al., 2015; Dao et al., 2016; Greer R.L. et al., 2016). It might not be surprising that the negative correlation between *A. muciniphila* and glucose levels was detected as one of the top ranked edges in our unbiased transkingdom network, thus, providing extra confidence for our results about less investigated bacteria inferred in our analyses.

Our predictions provide insights into host-microbial interactions. For instance, our result of *Bacteroides uniformis* being correlated with hepatic G6pase and Fxr might indicate potential mechanisms by which this bacterium improves glucose tolerance (Gauffin Cano et al., 2012). Also, it was shown that colonization with *Bacteroides thetaiotaomicron* makes mice leaner comparing controls despite similar levels of food consumption. The ability and preference of *Bacteroides thetaiotaomicron* and *Bacteroides ovatus* to utilize polysaccharide rich diet (McNulty et al., 2013) may explain these effects. However, our result of the negative correlation between abundance of these two bacteria and Pck1 (simple sugar forming gluconeogenic enzyme) may suggest the effect of these bacteria on liver gluconeogenesis. Similarly, *P. mirabilis* is predicted to have a negative interaction with GTT-AUC in our study, but shown to be positively correlated in rats with and without high fat diet (Lecomte et al., 2015). This disagreement may be explained by different physiological pathways dominating in the same bacterial species in different host that has been clearly shown for other bacteria (Oh et al., 2010). Overall, our study offers testable hypothesis regarding critical microbe-phenotype associations.

## Conclusion

We show that antibiotics alter systemic glucose metabolism in lean mice. In addition to reporting changes in the microbiota, expression of key genes from the glucose and bile acid metabolism pathways, and concomitant systemic metabolic measures, we delineate potential mechanisms by which microbes mediate these effects. While there is a general understanding of the different players and mechanisms of microbiome-mediated regulation of the glycemic response (Tremaroli and Backhed, 2012; Devaraj et al., 2013; Cani et al., 2014; Hartstra et al., 2015; Janssen and Kersten, 2015; Parekh et al., 2015; Sanz et al., 2015; Boulange et al., 2016; Marchesi et al., 2016; Stenman et al., 2016; Suez et al., 2016), a lot remains to be understood, especially in terms of identifying the precise pathways operating in host-microbiome interactions. Overall, our data strongly suggests that antibiotics affect systemic glucose metabolism via shaping gut microbial communities and consequently regulating gene expression programs in intestine and liver. Yet, treatment of germfree mice with antibiotics as well as colonization of germ-free mice with antibiotic modified microbiota are required to fully support above statement. Also, it is doubtful that different antibiotics use the same mechanisms of gene expression and microbiota changes to affect systemic glucose tolerance, and the limited number of samples per group makes it difficult to obtain antibiotic-specific mechanisms. Furthermore, while the taxonomical assignments of 16S rRNA sequencing of current study present natural challenges, further studies employing shotgun metagenomics sequencing will allow to overcome this limitation. Finally, our experimental design followed by a data-driven, systems biology approach of network analysis offers consistent and statistically significant interactions that may be integral in mediating the host-microbiome communication. Furthermore, this approach is a useful hypothesis generating strategy and future experimentation can help investigate the distinct mechanisms in the different antibiotics and eventually lead to personalized medicine (Zmora et al., 2016).

## Author Contributions

AM and NS conceived the original idea, designed and supervised the experiments, analyses, and writing. RG conceived the original idea, designed and performed the experiments, and supervised the writing. RR designed and performed the analyses, and drafted the manuscript. XD performed the analyses. JW, MG, and KD performed the experiments and analysis, respectively. All authors wrote the manuscript, read and approved the final draft submitted.

## Conflict of Interest Statement

The authors declare that the research was conducted in the absence of any commercial or financial relationships that could be construed as a potential conflict of interest.

## Supplementary Material

FIGURE S1 Dot plots with mean and error bars showing **(A)** body weight or **(B–E)** expression of the phenotypes across the different groups. The red and blue colors indicate experiments one and two, respectively. These phenotypes did not show statistically significant differences upon antibiotics treatment compared to untreated control mice.

FIGURE S2 Boxplots showing the Shannon diversity index. Asterisk indicate statistically significant differences upon antibiotics treatment compared to untreated control mice: same direction of (abx/control) fold change in both experiments, individual *p*-value < 2% in each experiment, Fisher’s combined *p*-value < 0.1% and FDR < 0.1%.

FIGURE S3 PCoA plot showing weighted UniFrac distance for cecal microbiota in the control and antibiotics treated mice. Each circle indicates a sample.

FIGURE S4 Taxonomic plots showing bacterial abundance across the different groups at the order level.

FIGURE S5 Taxonomic plots showing bacterial abundance across the different samples at the phylum level.

FIGURE S6 Taxonomic plots showing bacterial abundance across the different samples at the order level.

TABLE S1 Primers for the genes tested in this study.

TABLE S2 Alpha diversity metrics on the unrarefied and rarefied OTU tables in the two experiments.

TABLE S3 Levels of Firmicutes and Bacteroidetes in the two experiments. Asterisks indicate parameters that show statistically significant differences upon antibiotics treatment compared to untreated control mice: same direction of (abx/control) fold change in both experiments, individual *p*-value < 20% in each experiment, Fisher’s combined *p*-value < 5% and FDR < 10%.

TABLE S4 The ID, Greengenes taxonomy, and the median frequency per group for the OTUs in the same order as of the heatmap.

## Abbreviations

AUC: area under the curve
Fgf15: fibroblast growth factor 15
Fxr: farsenoid x receptor
G6pase: glucose 6-phosphatase
Glut1: glucose transporter 1
GTT: glucose tolerance test
Hk1: hexokinase 1
Hk2: hexokinase 2
Insr: insulin receptor
Pck1: phosphoenolpyruvate carboxykinase 1
Shp: small heterodimer partner
Tgr5: G protein-coupled bile acid receptor 1.

## References

[B1] Altschul S. F. Madden T. L. Schaffer A. A. Zhang J. Zhang Z. Miller W. (1997). Gapped BLAST and PSI-BLAST: a new generation of protein database search programs. *Nucleic Acids Res.* 25 3389–3402. 10.1093/nar/25.17.3389 9254694 PMC146917

[B2] Anhe F. F. Roy D. Pilon G. Dudonne S. Matamoros S. Varin T. V. (2015). A polyphenol-rich cranberry extract protects from diet-induced obesity, insulin resistance and intestinal inflammation in association with increased Akkermansia spp. population in the gut microbiota of mice. *Gut* 64 872–883. 10.1136/gutjnl-2014-307142 25080446

[B3] Assenov Y. Ramirez F. Schelhorn S. E. Lengauer T. Albrecht M. (2008). Computing topological parameters of biological networks. *Bioinformatics* 24 282–284. 10.1093/bioinformatics/btm554 18006545

[B4] Boulange C. L. Neves A. L. Chilloux J. Nicholson J. K. Dumas M. E. (2016). Impact of the gut microbiota on inflammation, obesity, and metabolic disease. *Genome Med.* 8:42. 10.1186/s13073-016-0303-2 27098727 PMC4839080

[B5] Caesar R. Reigstad C. S. Backhed H. K. Reinhardt C. Ketonen M. Lunden G. O. (2012). Gut-derived lipopolysaccharide augments adipose macrophage accumulation but is not essential for impaired glucose or insulin tolerance in mice. *Gut* 61 1701–1707. 10.1136/gutjnl-2011-301689 22535377 PMC3505865

[B6] Cani P. D. Bibiloni R. Knauf C. Waget A. Neyrinck A. M. Delzenne N. M. (2008). Changes in gut microbiota control metabolic endotoxemia-induced inflammation in high-fat diet-induced obesity and diabetes in mice. *Diabetes Metab. Res. Rev.* 57 1470–1481. 10.2337/db07-1403 18305141

[B7] Cani P. D. Geurts L. Matamoros S. Plovier H. Duparc T. (2014). Glucose metabolism: focus on gut microbiota, the endocannabinoid system and beyond. *Diabetes Metab.* 40 246–257. 10.1016/j.diabet.2014.02.004 24631413

[B8] Caporaso J. G. Kuczynski J. Stombaugh J. Bittinger K. Bushman F. D. Costello E. K. (2010). QIIME allows analysis of high-throughput community sequencing data. *Nat. Methods* 7 335–336. 10.1038/nmeth.f.303 20383131 PMC3156573

[B9] Caporaso J. G. Lauber C. L. Walters W. A. Berg-Lyons D. Huntley J. Fierer N. (2012). Ultra-high-throughput microbial community analysis on the Illumina HiSeq and MiSeq platforms. *ISME J.* 6 1621–1624. 10.1038/ismej.2012.8 22402401 PMC3400413

[B10] Carvalho B. M. Guadagnini D. Tsukumo D. M. L. Schenka A. A. Latuf-Filho P. Vassallo J. (2012). Modulation of gut microbiota by antibiotics improves insulin signalling in high-fat fed mice. *Diabetologia* 55 2823–2834. 10.1007/s00125-012-2648-4 22828956

[B11] Chakraborti C. K. (2015). New-found link between microbiota and obesity. *World J. Gastrointest. Pathophysiol.* 6 110–119. 10.4291/wjgp.v6.i4.110 26600968 PMC4644874

[B12] Chiang J. Y. L. (2013). “Bile acid metabolism and signaling,” in *Comprehensive Physiology*, ed. Pollock D. M. (Hoboken, NJ: John Wiley & Sons, Inc.). 10.1002/cphy.c120023 PMC4422175 23897684

[B13] Dao M. C. Everard A. Aron-Wisnewsky J. Sokolovska N. Prifti E. Verger E. O. (2016). *Akkermansia muciniphila* and improved metabolic health during a dietary intervention in obesity: relationship with gut microbiome richness and ecology. *Gut* 65 426–436. 10.1136/gutjnl-2014-308778 26100928

[B14] De Fabiani E. Mitro N. Gilardi F. Caruso D. Galli G. Crestani M. (2003). Coordinated control of cholesterol catabolism to bile acids and of gluconeogenesis via a novel mechanism of transcription regulation linked to the fasted-to-fed cycle. *J. Biol. Chem.* 278 39124–39132. 10.1074/jbc.M305079200 12865425

[B15] De La Cochetiere M. F. Durand T. Lepage P. Bourreille A. Galmiche J. P. Dore J. (2005). Resilience of the dominant human fecal microbiota upon short-course antibiotic challenge. *J. Clin. Microbiol.* 43 5588–5592. 10.1128/JCM.43.11.5588-5592.2005 16272491 PMC1287787

[B16] De Vadder F. Kovatcheva-Datchary P. Zitoun C. Duchampt A. Backhed F. Mithieux G. (2016). Microbiota-produced succinate improves glucose homeostasis via intestinal gluconeogenesis. *Cell Metab.* 24 151–157. 10.1016/j.cmet.2016.06.013 27411015

[B17] DeSantis T. Z. Hugenholtz P. Larsen N. Rojas M. Brodie E. L. Keller K. (2006). Greengenes, a chimera-checked 16S rRNA gene database and workbench compatible with ARB. *Appl. Environ. Microbiol.* 72 5069–5072. 10.1128/AEM.03006-05 16820507 PMC1489311

[B18] Dethlefsen L. Huse S. Sogin M. L. Relman D. A. (2008). The pervasive effects of an antibiotic on the human gut microbiota, as revealed by deep 16S rRNA sequencing. *PLOS Biol.* 6:e280. 10.1371/journal.pbio.0060280 19018661 PMC2586385

[B19] Dethlefsen L. Relman D. A. (2011). Incomplete recovery and individualized responses of the human distal gut microbiota to repeated antibiotic perturbation. *Proc. Natl. Acad. Sci. U.S.A.* 108(Suppl._1), 4554–4561. 10.1073/pnas.1000087107 20847294 PMC3063582

[B20] Devaraj S. Hemarajata P. Versalovic J. (2013). The human gut microbiome and body metabolism: implications for obesity and diabetes. *Clin. Chem.* 59 617–628. 10.1373/clinchem.2012.187617 23401286 PMC3974587

[B21] Dong X. Yambartsev A. Ramsey S. A. Thomas L. D. Shulzhenko N. Morgun A. (2015). Reverse enGENEering of regulatory networks from big data: a roadmap for biologists. *Bioinform. Biol. Insights* 9 61–74. 10.4137/BBI.S12467 25983554 PMC4415676

[B22] Duran-Sandoval D. Mautino G. Martin G. Percevault F. Barbier O. Fruchart J. C. (2004). Glucose regulates the expression of the farnesoid X receptor in liver. *Diabetes* 53 890–898. 10.2337/diabetes.53.4.890 15047603

[B23] Edgar R. C. (2010). Search and clustering orders of magnitude faster than BLAST. *Bioinformatics* 26 2460–2461. 10.1093/bioinformatics/btq461 20709691

[B24] Everard A. Belzer C. Geurts L. Ouwerkerk J. P. Druart C. Bindels L. B. (2013). Cross-talk between *Akkermansia muciniphila* and intestinal epithelium controls diet-induced obesity. *Proc. Natl. Acad. Sci. U.S.A.* 110 9066–9071. 10.1073/pnas.1219451110 23671105 PMC3670398

[B25] Fisher R. A. (1932). “Statistical methods for research workers,” in *Biological Monographs and Manuals*, 5 Edn, eds Crew F. A. E. Cutler D. W. (Edinburgh: Oliver and Boyd).

[B26] Francino M. P. (2015). Antibiotics and the human gut microbiome: dysbioses and accumulation of resistances. *Front. Microbiol.* 6:1543. 10.3389/fmicb.2015.01543 26793178 PMC4709861

[B27] Fujisaka S. Ussar S. Clish C. Devkota S. Dreyfuss J. M. Sakaguchi M. (2016). Antibiotic effects on gut microbiota and metabolism are host dependent. *J. Clin. Invest.* 126 4430–4443. 10.1172/JCI86674 27775551 PMC5127688

[B28] Gauffin Cano P. Santacruz A. Moya A. Sanz Y. (2012). Bacteroides uniformis CECT 7771 ameliorates metabolic and immunological dysfunction in mice with high-fat-diet induced obesity. *PLOS ONE* 7:e41079. 10.1371/journal.pone.0041079 22844426 PMC3406031

[B29] Gower J. (1998). “Principal coordinate analysis,” in *Encyclopedia of Biostatistics*, eds Coulton T. Armitage P. (Hoboken, NJ: John Wiley and Sons Inc.), 3514–3518.

[B30] Greer R. Dong X. Morgun A. Shulzhenko N. (2016). Investigating a holobiont: microbiota perturbations and transkingdom networks. *Gut Microbes* 7 126–135. 10.1080/19490976.2015.1128625 26979110 PMC4856449

[B31] Greer R. L. Dong X. Moraes A. C. Zielke R. A. Fernandes G. R. Peremyslova E. (2016). *Akkermansia muciniphila* mediates negative effects of IFNgamma on glucose metabolism. *Nat. Commun.* 7:13329. 10.1038/ncomms13329 27841267 PMC5114536

[B32] Greer R. L. Morgun A. Shulzhenko N. (2013). Bridging immunity and lipid metabolism by gut microbiota. *J. Allergy Clin. Immunol.* 132 253–263. 10.1016/j.jaci.2013.06.025 23905915

[B33] Hansen C. H. Krych L. Nielsen D. S. Vogensen F. K. Hansen L. H. Sorensen S. J. (2012). Early life treatment with vancomycin propagates *Akkermansia muciniphila* and reduces diabetes incidence in the NOD mouse. *Diabetologia* 55 2285–2294. 10.1007/s00125-012-2564-7 22572803

[B34] Hartstra A. V. Bouter K. E. Backhed F. Nieuwdorp M. (2015). Insights into the role of the microbiome in obesity and type 2 diabetes. *Diabetes Care* 38 159–165. 10.2337/dc14-0769 25538312

[B35] Holt J. A. Luo G. Billin A. N. Bisi J. McNeill Y. Y. Kozarsky K. F. (2003). Definition of a novel growth factor-dependent signal cascade for the suppression of bile acid biosynthesis. *Genes Dev.* 17 1581–1591. 10.1101/gad.1083503 12815072 PMC196131

[B36] Horikoshi M. Tang Y. (2016). *ggfortify: Data Visualization Tools for Statistical Analysis Results*. Available at: https://cran.r-project.org/web/packages/ggfortify/index.html

[B37] Human Microbiome Project Consortium (2012). A framework for human microbiome research. *Nature* 486 215–221. 10.1038/nature11209 22699610 PMC3377744

[B38] Hwang I. Park Y. J. Kim Y. R. Kim Y. N. Ka S. Lee H. Y. (2015). Alteration of gut microbiota by vancomycin and bacitracin improves insulin resistance via glucagon-like peptide 1 in diet-induced obesity. *FASEB J.* 29 2397–2411. 10.1096/fj.14-265983 25713030

[B39] Jakobsson H. E. Jernberg C. Andersson A. F. Sjolund-Karlsson M. Jansson J. K. Engstrand L. (2010). Short-term antibiotic treatment has differing long-term impacts on the human throat and gut microbiome. *PLOS ONE* 5:e9836. 10.1371/journal.pone.0009836 20352091 PMC2844414

[B40] Janssen A. W. Kersten S. (2015). The role of the gut microbiota in metabolic health. *FASEB J.* 29 3111–3123. 10.1096/fj.14-269514 25921831

[B41] Jernberg C. Lofmark S. Edlund C. Jansson J. K. (2007). Long-term ecological impacts of antibiotic administration on the human intestinal microbiota. *ISME J.* 1 56–66. 10.1038/ismej.2007.3 18043614

[B42] Jiang C. Xie C. Li F. Zhang L. Nichols R. G. Krausz K. W. (2015a). Intestinal farnesoid X receptor signaling promotes nonalcoholic fatty liver disease. *J. Clin. Invest.* 125 386–402. 10.1172/JCI76738 25500885 PMC4382255

[B43] Jiang C. Xie C. Lv Y. Li J. Krausz K. W. Shi J. (2015b). Intestine-selective farnesoid X receptor inhibition improves obesity-related metabolic dysfunction. *Nat. Commun.* 6:10166. 10.1038/ncomms10166 26670557 PMC4682112

[B44] Joyce S. A. Gahan C. G. (2014). The gut microbiota and the metabolic health of the host. *Curr. Opin. Gastroenterol.* 30 120–127. 10.1097/MOG.0000000000000039 24468803

[B45] Karlsson F. H. Tremaroli V. Nookaew I. Bergstrom G. Behre C. J. Fagerberg B. (2013). Gut metagenome in European women with normal, impaired and diabetic glucose control. *Nature* 498 99–103. 10.1038/nature12198 23719380

[B46] Kasai C. Sugimoto K. Moritani I. Tanaka J. Oya Y. Inoue H. (2015). Comparison of the gut microbiota composition between obese and non-obese individuals in a Japanese population, as analyzed by terminal restriction fragment length polymorphism and next-generation sequencing. *BMC Gastroenterol.* 15:100. 10.1186/s12876-015-0330-2 26261039 PMC4531509

[B47] Komsta L. (2011). *Outliers: Tests for Outliers*. Available at: http://www.r-project.org

[B48] Kong B. Wang L. Chiang J. Y. Zhang Y. Klaassen C. D. Guo G. L. (2012). Mechanism of tissue-specific farnesoid X receptor in suppressing the expression of genes in bile-acid synthesis in mice. *Hepatology* 56 1034–1043. 10.1002/hep.25740 22467244 PMC3390456

[B49] Larsson E. Tremaroli V. Lee Y. S. Koren O. Nookaew I. Fricker A. (2012). Analysis of gut microbial regulation of host gene expression along the length of the gut and regulation of gut microbial ecology through MyD88. *Gut* 61 1124–1131. 10.1136/gutjnl-2011-301104 22115825 PMC3388726

[B50] Lecomte V. Kaakoush N. O. Maloney C. A. Raipuria M. Huinao K. D. Mitchell H. M. (2015). Changes in gut microbiota in rats fed a high fat diet correlate with obesity-associated metabolic parameters. *PLOS ONE* 10:e0126931. 10.1371/journal.pone.0126931 25992554 PMC4436290

[B51] Li G. Guo G. L. (2015). Farnesoid X receptor, the bile acid sensing nuclear receptor, in liver regeneration. *Acta Pharm. Sin. B* 5 93–98. 10.1016/j.apsb.2015.01.005 26579433 PMC4629218

[B52] Lozupone C. Knight R. (2005). UniFrac: a new phylogenetic method for comparing microbial communities. *Appl. Environ. Microbiol.* 71 8228–8235. 10.1128/AEM.71.12.8228-8235.2005 16332807 PMC1317376

[B53] Ma K. Saha P. K. Chan L. Moore D. D. (2006). Farnesoid X receptor is essential for normal glucose homeostasis. *J. Clin. Invest.* 116 1102–1109. 10.1172/JCI25604 16557297 PMC1409738

[B54] Marchesi J. R. Adams D. H. Fava F. Hermes G. D. Hirschfield G. M. Hold G. (2016). The gut microbiota and host health: a new clinical frontier. *Gut* 65 330–339. 10.1136/gutjnl-2015-309990 26338727 PMC4752653

[B55] McDonald D. Price M. N. Goodrich J. Nawrocki E. P. DeSantis T. Z. Probst A. (2012). An improved Greengenes taxonomy with explicit ranks for ecological and evolutionary analyses of bacteria and archaea. *ISME J.* 6 610–618. 10.1038/ismej.2011.139 22134646 PMC3280142

[B56] McNulty N. P. Wu M. Erickson A. R. Pan C. Erickson B. K. Martens E. C. (2013). Effects of diet on resource utilization by a model human gut microbiota containing Bacteroides cellulosilyticus WH2, a symbiont with an extensive glycobiome. *PLOS Biol.* 11:e1001637. 10.1371/journal.pbio.1001637 23976882 PMC3747994

[B57] Membrez M. Blancher F. Jaquet M. Bibiloni R. Cani P. D. Burcelin R. G. (2008). Gut microbiota modulation with norfloxacin and ampicillin enhances glucose tolerance in mice. *FASEB J.* 22 2416–2426. 10.1096/fj.07-102723 18326786

[B58] Mikkelsen K. H. Allin K. H. Knop F. K. (2016). Effect of antibiotics on gut microbiota, glucose metabolism and body weight regulation: a review of the literature. *Diabetes. Obes. Metab.* 18 444–453. 10.1111/dom.12637 26818734

[B59] Mikkelsen K. H. Frost M. Bahl M. I. Licht T. R. Jensen U. S. Rosenberg J. (2015). Effect of antibiotics on gut microbiota, gut hormones and glucose metabolism. *PLOS ONE* 10:e0142352. 10.1371/journal.pone.0142352 26562532 PMC4643023

[B60] Modica S. Gadaleta R. M. Moschetta A. (2010). Deciphering the nuclear bile acid receptor FXR paradigm. *Nucl. Recept. Signal.* 8:e005. 10.1621/nrs.08005 21383957 PMC3049226

[B61] Morgulis A. Coulouris G. Raytselis Y. Madden T. L. Agarwala R. Schaffer A. A. (2008). Database indexing for production MegaBLAST searches. *Bioinformatics* 24 1757–1764. 10.1093/bioinformatics/btn322 18567917 PMC2696921

[B62] Morgun A. Dzutsev A. Dong X. Greer R. L. Sexton D. J. Ravel J. (2015). Uncovering effects of antibiotics on the host and microbiota using transkingdom gene networks. *Gut* 64 1732–1743. 10.1136/gutjnl-2014-308820 25614621 PMC5166700

[B63] Murri M. Leiva I. Gomez-Zumaquero J. M. Tinahones F. J. Cardona F. Soriguer F. (2013). Gut microbiota in children with type 1 diabetes differs from that in healthy children: a case-control study. *BMC Med.* 11:46. 10.1186/1741-7015-11-46 23433344 PMC3621820

[B64] Nguyen A. Bouscarel B. (2008). Bile acids and signal transduction: role in glucose homeostasis. *Cell. Signal.* 20 2180–2197. 10.1016/j.cellsig.2008.06.014 18634871

[B65] Nie Y. F. Hu J. Yan X. H. (2015). Cross-talk between bile acids and intestinal microbiota in host metabolism and health. *J. Zhejiang Univ. Sci. B* 16 436–446. 10.1631/jzus.B1400327 26055905 PMC4471595

[B66] Oh P. L. Benson A. K. Peterson D. A. Patil P. B. Moriyama E. N. Roos S. (2010). Diversification of the gut symbiont *Lactobacillus reuteri* as a result of host-driven evolution. *ISME J.* 4 377–387. 10.1038/ismej.2009.123 19924154

[B67] Pallav K. Dowd S. E. Villafuerte J. Yang X. Kabbani T. Hansen J. (2014). Effects of polysaccharopeptide from *Trametes versicolor* and amoxicillin on the gut microbiome of healthy volunteers: a randomized clinical trial. *Gut Microbes* 5 458–467. 10.4161/gmic.29558 25006989

[B68] Panda S. El khader I. Casellas F. Lopez Vivancos J. Garcia Cors M. Santiago A. (2014). Short-term effect of antibiotics on human gut microbiota. *PLOS ONE* 9:e95476. 10.1371/journal.pone.0095476 24748167 PMC3991704

[B69] Pang J. Rhodes D. H. Pini M. Akasheh R. T. Castellanos K. J. Cabay R. J. (2013). Increased adiposity, dysregulated glucose metabolism and systemic inflammation in Galectin-3 KO mice. *PLOS ONE* 8:e57915. 10.1371/journal.pone.0057915 23451284 PMC3579848

[B70] Parekh P. J. Balart L. A. Johnson D. A. (2015). The influence of the gut microbiome on obesity, metabolic syndrome and gastrointestinal disease. *Clin. Transl. Gastroenterol.* 6:e91. 10.1038/ctg.2015.16 26087059 PMC4816244

[B71] Pathak P. Liu H. Boehme S. Xie C. Krausz K. W. Gonzalez F. (2017). Farnesoid X receptor induces Takeda G-protein receptor 5 cross-talk to regulate bile acid synthesis and hepatic metabolism. *J. Biol. Chem.* 292 11055–11069. 10.1074/jbc.M117.784322 28478385 PMC5491788

[B72] Paulson J. N. Stine O. C. Bravo H. C. Pop M. (2013). Differential abundance analysis for microbial marker-gene surveys. *Nat. Methods* 10 1200–1202. 10.1038/nmeth.2658 24076764 PMC4010126

[B73] Perez-Cobas A. E. Gosalbes M. J. Friedrichs A. Knecht H. Artacho A. Eismann K. (2013). Gut microbiota disturbance during antibiotic therapy: a multi-omic approach. *Gut* 62 1591–1601. 10.1136/gutjnl-2012-303184 23236009 PMC3812899

[B74] Potthoff M. J. Boney-Montoya J. Choi M. He T. Sunny N. E. Satapati S. (2011). FGF15/19 regulates hepatic glucose metabolism by inhibiting the CREB-PGC-1alpha pathway. *Cell Metab.* 13 729–738. 10.1016/j.cmet.2011.03.019 21641554 PMC3131185

[B75] Prawitt J. Caron S. Staels B. (2011). Bile acid metabolism and the pathogenesis of type 2 diabetes. *Curr. Diab. Rep.* 11 160–166. 10.1007/s11892-011-0187-x 21431855 PMC3338411

[B76] Qin J. Li Y. Cai Z. Li S. Zhu J. Zhang F. (2012). A metagenome-wide association study of gut microbiota in type 2 diabetes. *Nature* 490 55–60. 10.1038/nature11450 23023125

[B77] Rakoff-Nahoum S. Paglino J. Eslami-Varzaneh F. Edberg S. Medzhitov R. (2004). Recognition of commensal microflora by toll-like receptors is required for intestinal homeostasis. *Cell* 118 229–241. 10.1016/j.cell.2004.07.002 15260992

[B78] Raymond F. Ouameur A. A. Deraspe M. Iqbal N. Gingras H. Dridi B. (2016). The initial state of the human gut microbiome determines its reshaping by antibiotics. *ISME J.* 10 707–720. 10.1038/ismej.2015.148 26359913 PMC4817689

[B79] Ritchie M. E. Phipson B. Wu D. Hu Y. F. Law C. W. Shi W. (2015). limma powers differential expression analyses for RNA-sequencing and microarray studies. *Nucleic Acids Res.* 43:e47. 10.1093/nar/gkv007 25605792 PMC4402510

[B80] Rodrigues R. R. Shulzhenko N. Morgun A. (2017). Transkingdom networks: a systems biology approach to identify causal members of host-microbiota interactions. (accepted). Available at: https://arxiv.org/pdf/1709.05701.pdf 10.1007/978-1-4939-8728-3_15 PMC6557635 30298258

[B81] Saeidi N. Meoli L. Nestoridi E. Gupta N. K. Kvas S. Kucharczyk J. (2013). Reprogramming of intestinal glucose metabolism and glycemic control in rats after gastric bypass. *Science* 341 406–410. 10.1126/science.1235103 23888041 PMC4068965

[B82] Sanz Y. Olivares M. Moya-Perez A. Agostoni C. (2015). Understanding the role of gut microbiome in metabolic disease risk. *Pediatr. Res.* 77 236–244. 10.1038/pr.2014.170 25314581

[B83] Sayin S. I. Wahlstrom A. Felin J. Jantti S. Marschall H. U. Bamberg K. (2013). Gut microbiota regulates bile acid metabolism by reducing the levels of tauro-beta-muricholic acid, a naturally occurring FXR antagonist. *Cell Metab.* 17 225–235. 10.1016/j.cmet.2013.01.003 23395169

[B84] Schneeberger M. Everard A. Gomez-Valades A. G. Matamoros S. Ramirez S. Delzenne N. M. (2015). *Akkermansia muciniphila* inversely correlates with the onset of inflammation, altered adipose tissue metabolism and metabolic disorders during obesity in mice. *Sci. Rep.* 5:16643. 10.1038/srep16643 26563823 PMC4643218

[B85] Shannon P. Markiel A. Ozier O. Baliga N. S. Wang J. T. Ramage D. (2003). Cytoscape: a software environment for integrated models of biomolecular interaction networks. *Genome Res.* 13 2498–2504. 10.1101/gr.1239303 14597658 PMC403769

[B86] Shin N. R. Lee J. C. Lee H. Y. Kim M. S. Whon T. W. Lee M. S. (2014). An increase in the Akkermansia spp. population induced by metformin treatment improves glucose homeostasis in diet-induced obese mice. *Gut* 63 727–735. 10.1136/gutjnl-2012-303839 23804561

[B87] Stenman L. K. Burcelin R. Lahtinen S. (2016). Establishing a causal link between gut microbes, body weight gain and glucose metabolism in humans - towards treatment with probiotics. *Benef. Microbes* 7 11–22. 10.3920/BM2015.0069 26565087

[B88] Suez J. Shapiro H. Elinav E. (2016). Role of the microbiome in the normal and aberrant glycemic response. *Clin. Nutr. Exp.* 6 59–73. 10.1016/j.yclnex.2016.01.001

[B89] Thomas C. Pellicciari R. Pruzanski M. Auwerx J. Schoonjans K. (2008). Targeting bile-acid signalling for metabolic diseases. *Nat. Rev. Drug Discov.* 7 678–693. 10.1038/nrd2619 18670431

[B90] Trabelsi M. S. Lestavel S. Staels B. Collet X. (2016). Intestinal bile acid receptors are key regulators of glucose homeostasis. *Proc. Nutr. Soc.* 76 192–202. 10.1017/S0029665116002834 27846919

[B91] Trauner M. Claudel T. Fickert P. Moustafa T. Wagner M. (2010). Bile acids as regulators of hepatic lipid and glucose metabolism. *Dig. Dis.* 28 220–224. 10.1159/000282091 20460915

[B92] Tremaroli V. Backhed F. (2012). Functional interactions between the gut microbiota and host metabolism. *Nature* 489 242–249. 10.1038/nature11552 22972297

[B93] Ubeda C. Taur Y. Jenq R. R. Equinda M. J. Son T. Samstein M. (2010). Vancomycin-resistant Enterococcus domination of intestinal microbiota is enabled by antibiotic treatment in mice and precedes bloodstream invasion in humans. *J. Clin. Invest.* 120 4332–4341. 10.1172/JCI43918 21099116 PMC2993598

[B94] Utzschneider K. M. Kratz M. Damman C. J. Hullarg M. (2016). Mechanisms linking the gut microbiome and glucose metabolism. *J. Clin. Endocrinol. Metab.* 101 1445–1454. 10.1210/jc.2015-4251 26938201 PMC4880177

[B95] Vazquez-Baeza Y. Pirrung M. Gonzalez A. Knight R. (2013). EMPeror: a tool for visualizing high-throughput microbial community data. *Gigascience* 2:16. 10.1186/2047-217X-2-16 24280061 PMC4076506

[B96] Warnes G. R. Bolker B. Bonebakker L. Gentleman R. Liaw W. H. A. Lumley T. (2016). *gplots: Various R Programming Tools for Plotting Data*. Available at: https://CRAN.R-project.org/package=gplots

[B97] Wickham H. (2009). *ggplot2: Elegant Graphics for Data Analysis*. New York, NY: Springer-Verlag 10.1007/978-0-387-98141-3

[B98] Wu H. Tremaroli V. Backhed F. (2015). Linking microbiota to human diseases: a systems biology perspective. *Trends Endocrinol. Metab.* 26 758–770. 10.1016/j.tem.2015.09.011 26555600

[B99] Yamagata K. Daitoku H. Shimamoto Y. Matsuzaki H. Hirota K. Ishida J. (2004). Bile acids regulate gluconeogenic gene expression via small heterodimer partner-mediated repression of hepatocyte nuclear factor 4 and Foxo1. *J. Biol. Chem.* 279 23158–23165. 10.1074/jbc.M314322200 15047713

[B100] Yambartsev A. Perlin M. A. Kovchegov Y. Shulzhenko N. Mine K. L. Dong X. (2016). Unexpected links reflect the noise in networks. *Biol. Direct* 11:52. 10.1186/s13062-016-0155-0 27737689 PMC5480421

[B101] Zhang X. Shen D. Fang Z. Jie Z. Qiu X. Zhang C. (2013). Human gut microbiota changes reveal the progression of glucose intolerance. *PLOS ONE* 8:e71108. 10.1371/journal.pone.0071108 24013136 PMC3754967

[B102] Zhang Y. Lee F. Y. Barrera G. Lee H. Vales C. Gonzalez F. J. (2006). Activation of the nuclear receptor FXR improves hyperglycemia and hyperlipidemia in diabetic mice. *Proc. Natl. Acad. Sci. U.S.A.* 103 1006–1011. 10.1073/pnas.0506982103 16410358 PMC1347977

[B103] Zhou M. Luo J. Chen M. Yang H. Learned R. M. DePaoli A. M. (2017). Mouse species-specific control of hepatocarcinogenesis and metabolism by FGF19/FGF15. *J. Hepatol.* 66 1182–1192. 10.1016/j.jhep.2017.01.027 28189755

[B104] Zmora N. Zeevi D. Korem T. Segal E. Elinav E. (2016). Taking it personally: personalized utilization of the human microbiome in health and disease. *Cell Host Microbe* 19 12–20. 10.1016/j.chom.2015.12.016 26764593

