# Supplementary figures and images for: Antibiotic-Induced Alterations in Gut Microbiota Are Associated with Changes in Glucose Metabolism in Healthy Mice
Source: Front Microbiol. 2017 Nov 22;8:2306. doi: 10.3389/fmicb.2017.02306 (PMC5702803; doi:10.3389/fmicb.2017.02306)

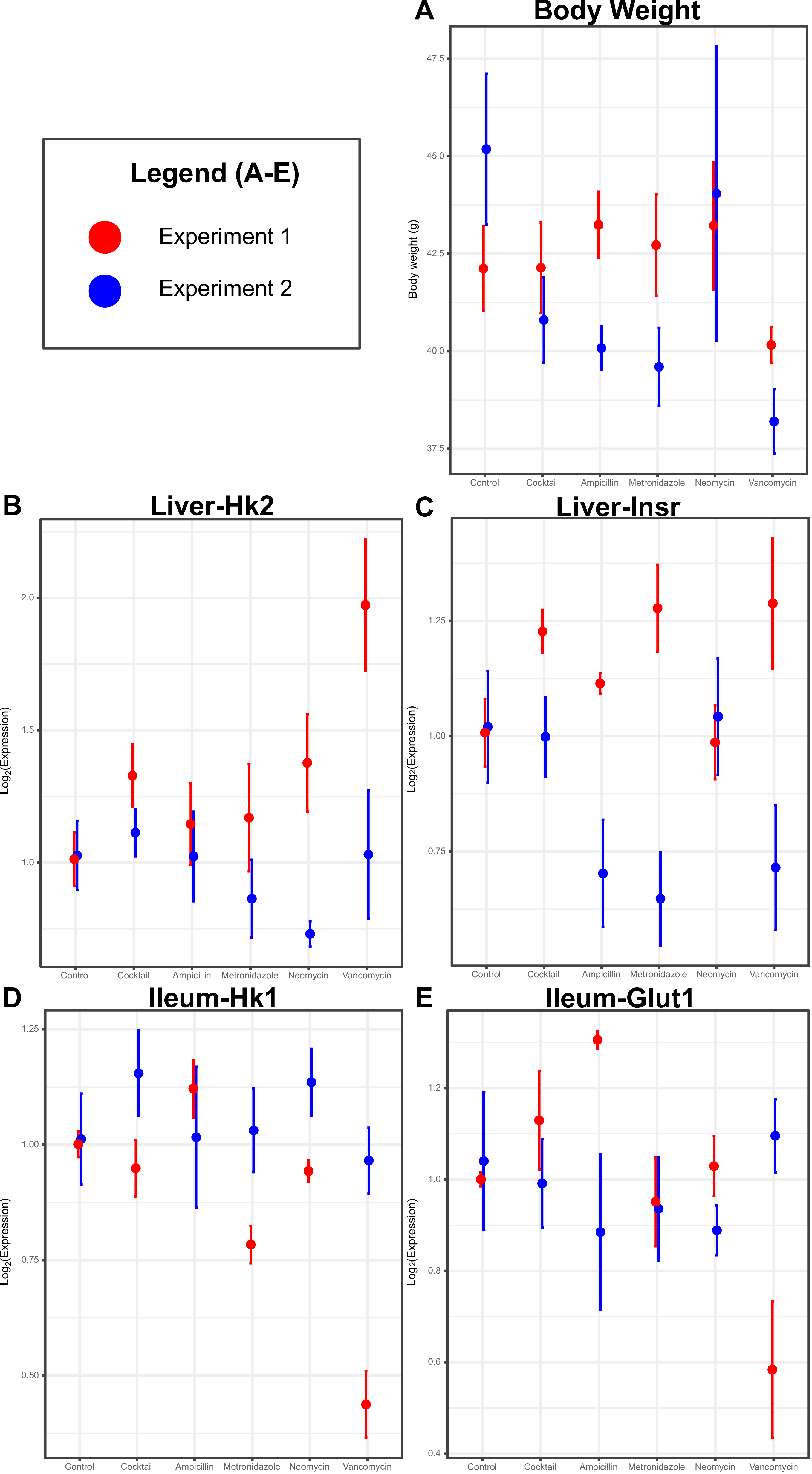

Supplement: FIGURE S1 — Dot plots with mean and error bars showing (A) body weight or (B–E) expression of the phenotypes across the different groups. The red and blue colors indicate experiments one and two, respectively. These phenotypes did not show statistically significant differences upon antibiotics treatment compared to untreated control mice. [file Image_1.TIFF]

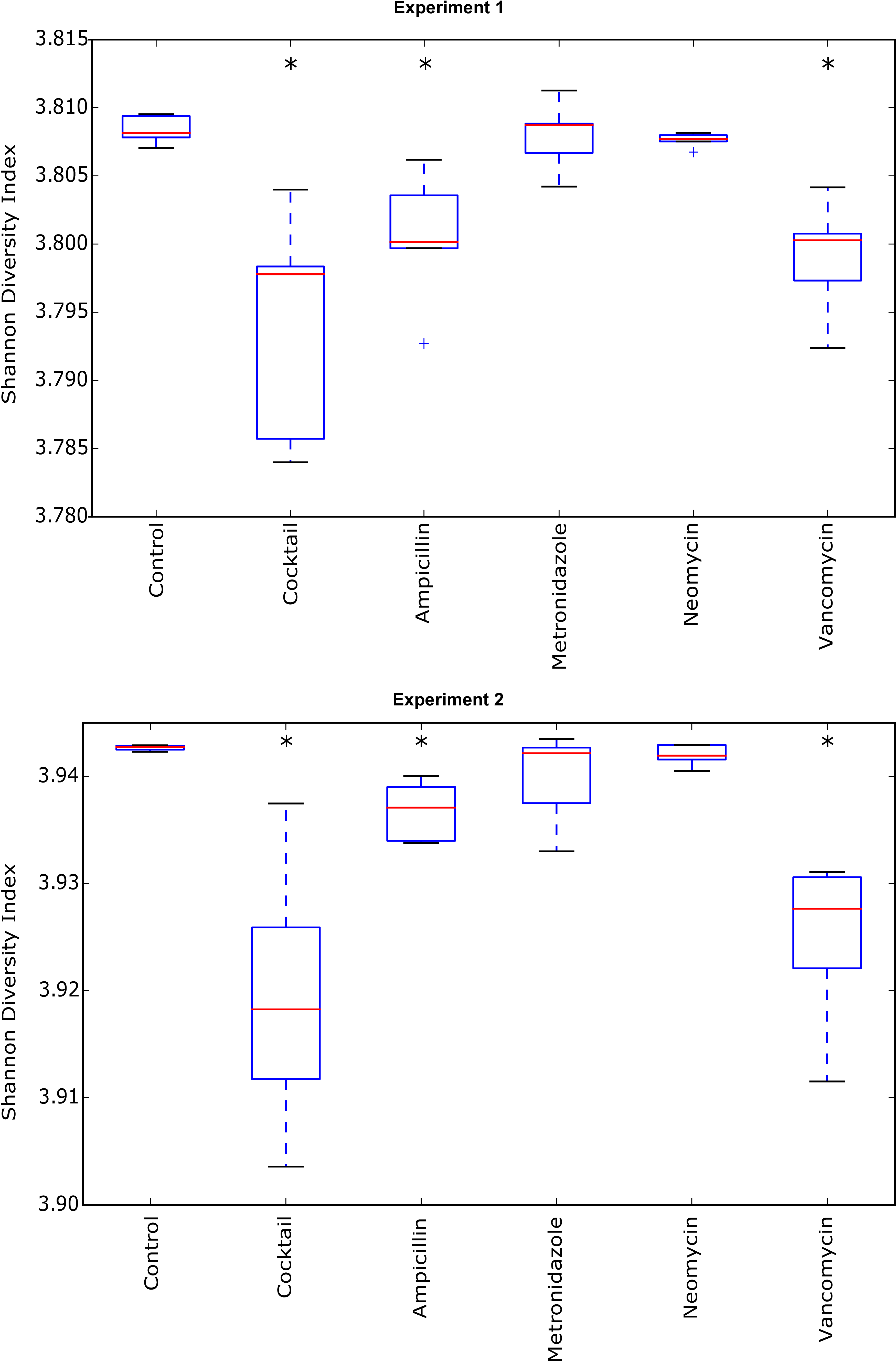

Supplement: FIGURE S2 — Boxplots showing the Shannon diversity index. Asterisk indicate statistically significant differences upon antibiotics treatment compared to untreated control mice: same direction of (abx/control) fold change in both experiments, individual p-value < 2% in each experiment, Fisher’s combined p-value < 0.1% and FDR < 0.1%. [file Image_2.TIFF]

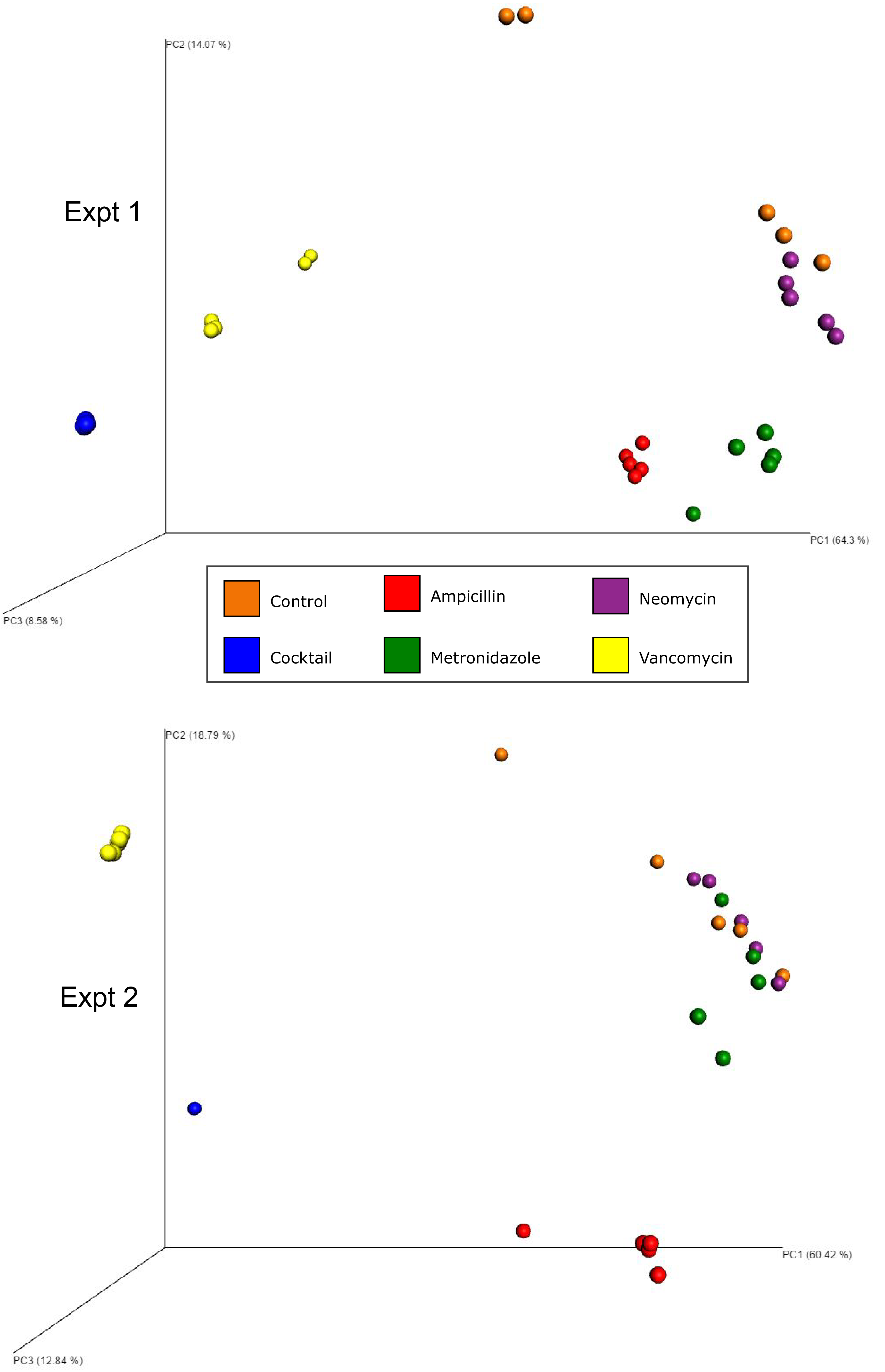

Supplement: FIGURE S3 — PCoA plot showing weighted UniFrac distance for cecal microbiota in the control and antibiotics treated mice. Each circle indicates a sample. [file Image_3.TIFF]

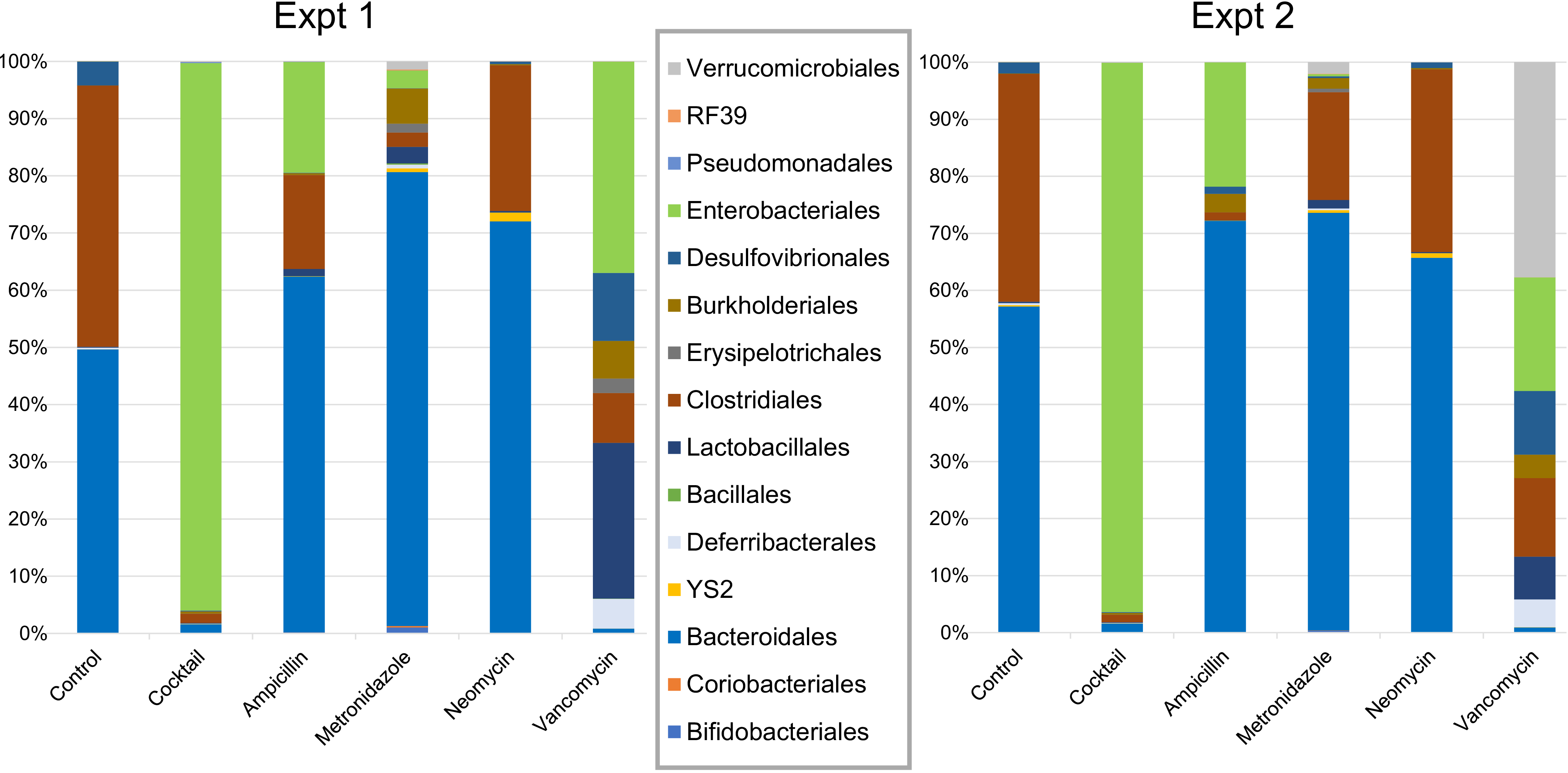

Supplement: FIGURE S4 — Taxonomic plots showing bacterial abundance across the different groups at the order level. [file Image_4.TIFF]

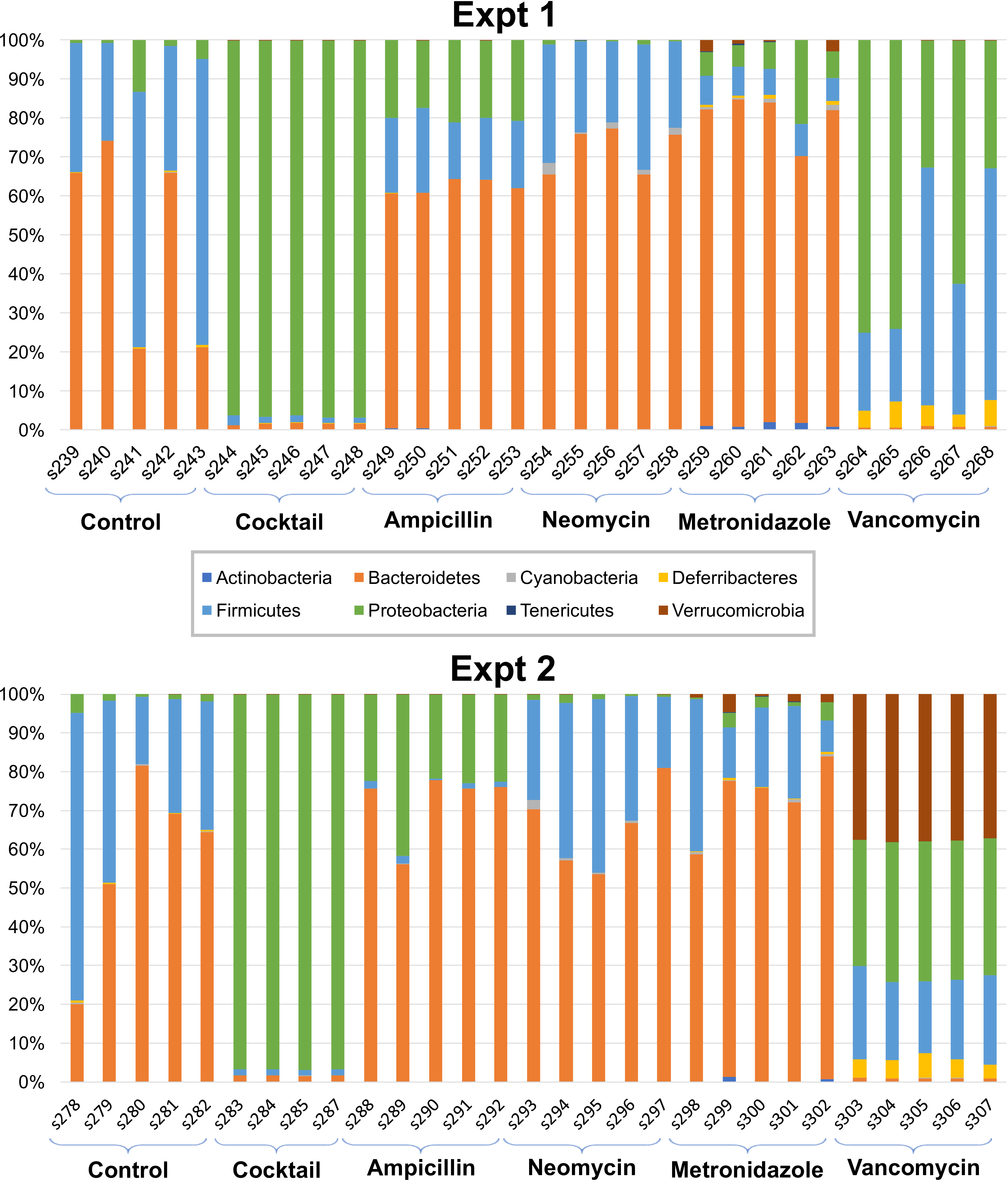

Supplement: FIGURE S5 — Taxonomic plots showing bacterial abundance across the different samples at the phylum level. [file Image_5.TIFF]

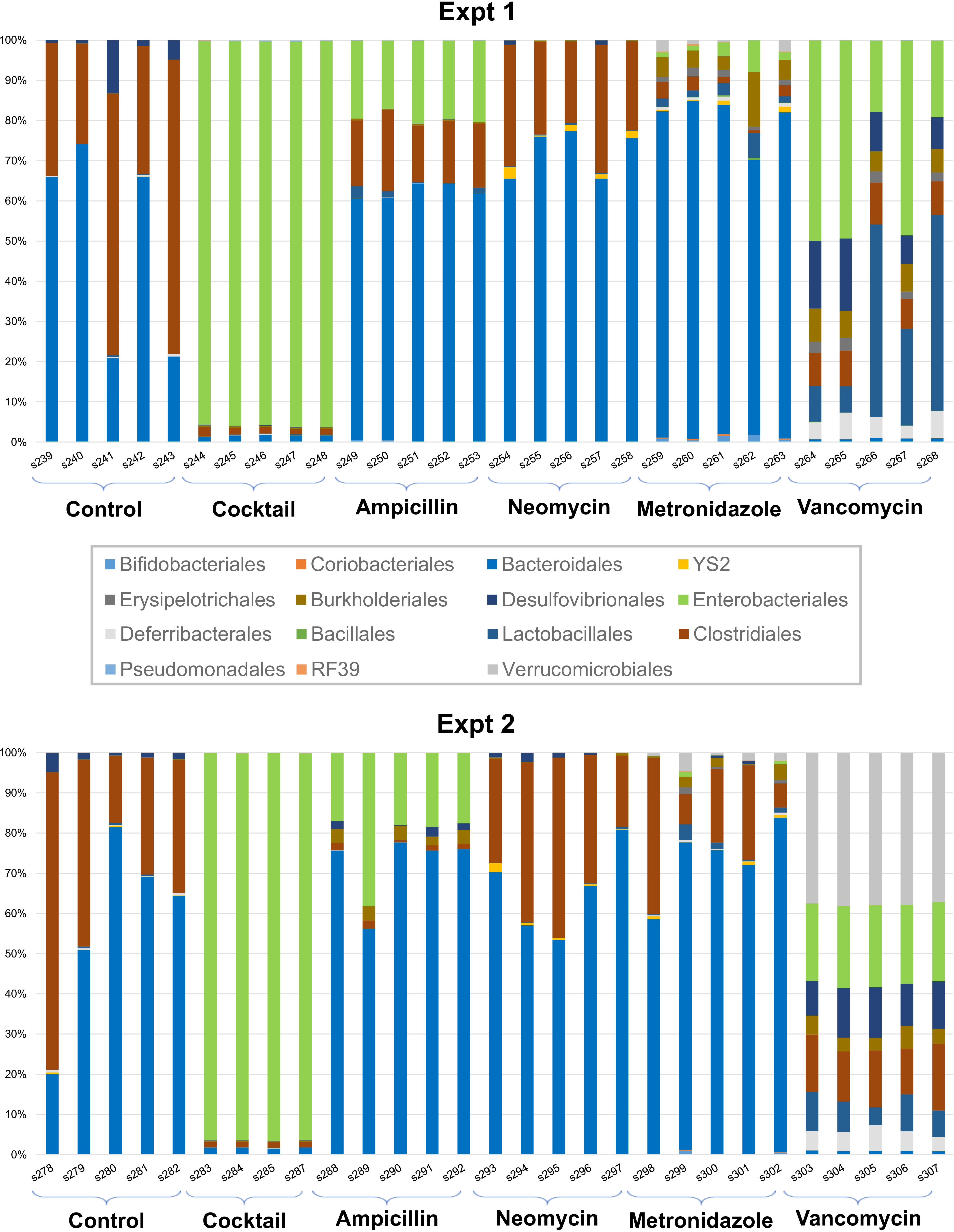

Supplement: FIGURE S6 — Taxonomic plots showing bacterial abundance across the different samples at the order level. [file Image_6.TIFF]
